# European Headache Federation guideline on idiopathic intracranial hypertension

**DOI:** 10.1186/s10194-018-0919-2

**Published:** 2018-10-08

**Authors:** Jan Hoffmann, Susan P Mollan, Koen Paemeleire, Christian Lampl, Rigmor H Jensen, Alexandra J Sinclair

**Affiliations:** 1 Basic and Clinical Neuroscience, Institute of Psychiatry, Psychology and Neuroscience, Wellcome Foundation Building, Denmark Hill Campus, King’s College London, London, SE5 9PJ UK; 2 Birmingham Neuro-Ophthalmology, University Hospitals Birmingham NHS Foundation Trust, Queen Elizabeth Hospital, Birmingham, UK; 3 Department of Neurology, Ghent University Hospital, Ghent, Belgium; 4 Headache Medical Centre, Seilerstaette Linz, Ordensklinikum Linz, Barmherzige Schwestern, Linz, Austria; 5 Danish Headache Center, Department of Neurology, Rigshospitalet-Glostrup, University of Copenhagen, Glostrup, Denmark; 6 Metabolic Neurology, Institute of Metabolism and Systems Research, University of Birmingham, Edgbaston, UK

## Abstract

**Background:**

Idiopathic Intracranial Hypertension (IIH) is characterized by an elevation of intracranial pressure (ICP no identifiable cause. The aetiology remains largely unknown, however observations made in a number of recent clinical studies are increasing the understanding of the disease and now provide the basis for evidence-based treatment strategies.

**Methods:**

The Embase, CDSR, CENTRAL, DARE and MEDLINE databases were searched up to 1st June 2018. We analyzed randomized controlled trials and systematic reviews that investigate IIH.

**Results:**

Diagnostic uncertainty, headache morbidity and visual loss are among the highest concerns of clinicians and patients in this disease area. Research in this field is infrequent due to the rarity of the disease and the lack of understanding of the underlying pathology.

**Conclusions:**

This European Headache Federation consensus paper provides evidence-based recommendations and practical advice on the investigation and management of IIH.

**Electronic supplementary material:**

The online version of this article (10.1186/s10194-018-0919-2) contains supplementary material, which is available to authorized users.

## Objective

Idiopathic Intracranial Hypertension (IIH) is characterized by an elevation of intracranial pressure (ICP) with no identifiable cause [1]. Despite the fact that its aetiology remains largely unknown, the observations made in a significant number of recent clinical studies and the resulting increase in the understanding of its clinical picture have led to modifications in its diagnostic classification and provide the basis for evidence-based treatment strategies. This consensus paper is based on the current literature on diagnosis and treatment of IIH and provides evidence-based recommendations on its treatment where randomized-controlled trials are available.

## Background

**Fig. 1:**
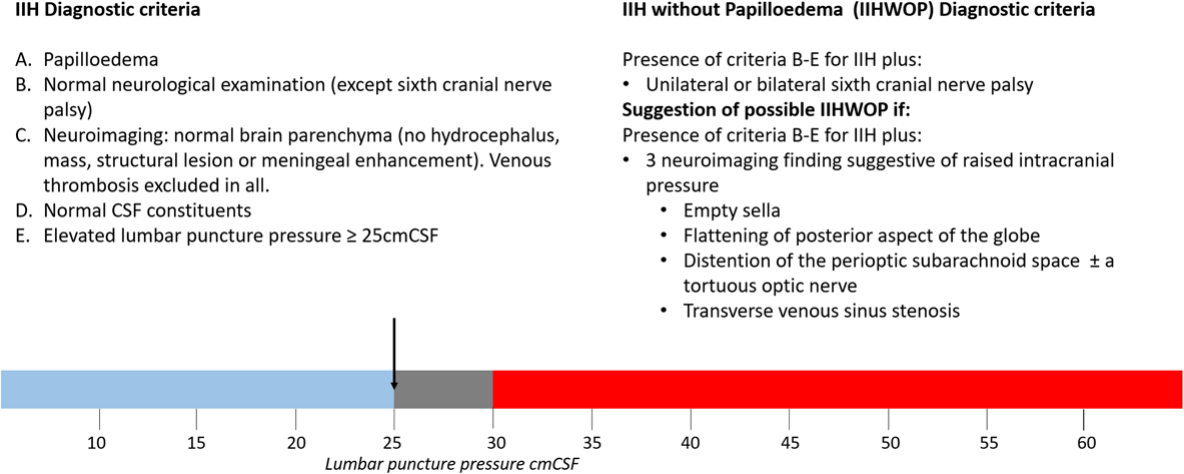
Diagnostic criteria for IIH (Friedman criteria). Diagnostic criteria for IIH and IIH without papilloedema. Infogram demonstrating the “grey zone” in which LP pressure is normal in some individuals but can indicate pathologically raised ICP in some. Measurements in the grey zone need to be interpreted with caution and patients must fit the other criteria for IIH for a diagnosis to be confirmed

The entire clinical syndrome of IIH is defined in the diagnostic criteria established by Friedman et al. [2] (Fig. 1) whereas the associated headache is defined in the Headache Classification of the International Headache Society (IHS) [3] (Table 1). The term ‘pseudotumor cerebri’, in the past commonly used as a synonym for IIH, is now used as an umbrella term that describes the chronic elevation of ICP regardless of its aetiology and further subdivides in the primary (IIH) and secondary forms [2]. The remit of this manuscript will exclusively focus on the diagnostic and therapeutic algorithm of primary IIH.

**Table 1:** Diagnostic criteria for IIH-related headache according to the International Classification of Headache Disorders (ICHD-3)

A. New headache, or a significant worsening of a pre-existing headache, fulfilling criterion C B. Both of the following: 1. idiopathic intracranial hypertension (IIH) has been diagnosed 2. cerebrospinal fluid (CSF) pressure exceeds 250 mm CSF (or 280 mm CSF in obese children) C. Either or both of the following: 1. headache has developed or significantly worsened in temporal relation to the IIH, or led to its discovery 2. headache is accompanied by either or both of the following: a) pulsatile tinnitus b) papilloedema D. Not better accounted for by another ICHD-3 diagnosis	

IIH is a disorder that mainly affects obese women of childbearing age. Its prevalence has been estimated between 0.5–2 per 100,000 of the general population [4]. However, due to the lack of awareness of the clinical syndrome as well as its potential similarity to primary headaches including migraine, it has been suggested that the disorder may be underdiagnosed [5]. In contrast, given the strong association between obesity and elevated ICP, it can be expected that the increasing obesity in the general population will increase the prevalence as well as the socioeconomic burden of the disease [1, 6]. This outlook underlines the importance of clear diagnostic criteria that allow an accurate diagnostic algorithm as well as recommending effective management strategies.

## Clinical symptoms and diagnostic algorithm

### Clinical picture

#### Headache

Headache, present in up to 90% of IIH patients, is commonly the primary symptom leading IIH patients to seek medical advice [7, 8]. Headache is also the key factor driving reduced quality of life in IIH [9]. The features of IIH-related headache vary substantially and in the context of a limited amount of clinical studies that aim at characterizing them, the IHS-criteria remain relatively unspecific in their description. Patients commonly describe their headache as pressing, explosive with a frontal, retroorbital localization [3, 10]. Frequently the headache has a migraine phenotype and overuse of analgesic is observed in over a third of IIH patients [11]. Phenotypic similarities may hamper its distinction from migraine and other headaches [7, 8, 12–17]. In order to establish the diagnosis of IIH it is required that the causality between the clinical symptoms and elevated ICP is demonstrated by a temporal relationship between headache onset and the identification of elevated ICP or an alleviation after a reduction of ICP. However, one study reported that over 20% of patients with other headache disorders also improve after lumbar puncture (LP) [18]. The second edition of the International Headache Classification (ICHD-2) detailed headache alleviation after pressure reduction was a required diagnostic criterion of IIH, however in the recent ICHD-3 criteria this criterion has been removed as headache duration can vary substantially with almost two thirds of IIH-patients complaining of persisting chronic headache despite a normalization of ICP [3, 10, 18, 19]. However, the precise onset of increased ICP and the related headache are difficult to establish, so the preliminary version of the present diagnostic ICHD-3 criteria (ICHD-3 beta) for IIH-headache have been field tested in a recent study and more sensitive and specific criteria have been suggested [18].

#### Ophthalmic features

Bilateral disc swelling, termed papilloedema when it is caused by raised ICP, is a cardinal feature of IIH and on examination it can be asymmetrical in 4% of cases [7, 20–25]. Examination of the eye can be challenging and in case of diagnostic uncertainty, papilloedema should be confirmed by an experienced ophthalmologist. Exclusion of pseudopapilloedema is recommended to prevent unnecessary investigations and procedures. These include measurements of intraocular pressure, ruling out hypotony, and critical examination of the optic nerve as small hypermetropic discs, titled myopic discs, vitreous traction and disc drusen can all be mistaken for papilloedema. There is a high frequency (40%) of diagnostic errors in IIH, with the main cause being an incorrect ophthalmic examination [26].

Raised ICP can lead to a number of visual symptoms including transient visual obscurations, visual blurring and double vision. All IIH patients with active papilloedema need close ophthalmological monitoring to evaluate the visual function and assess the risk of visual loss as in some the visual disturbances are progressive and may lead to prolonged ischemia of the optic nerve head resulting in complete and irreversible sight loss secondary to optic atrophy. The tests of visual function are important as no correlation between headache frequency and the degree of papilloedema has been demonstrated [27]. Data from a recent randomized-controlled clinical trial with acetazolamide for the treatment of IIH, the IIH Treatment Trial (IIHTT), revealed that higher-grade papilloedema and a significant loss in visual acuity at presentation are associated with a higher risk of progression to visual field loss despite adequate treatment [28].

**Fig. 2:**
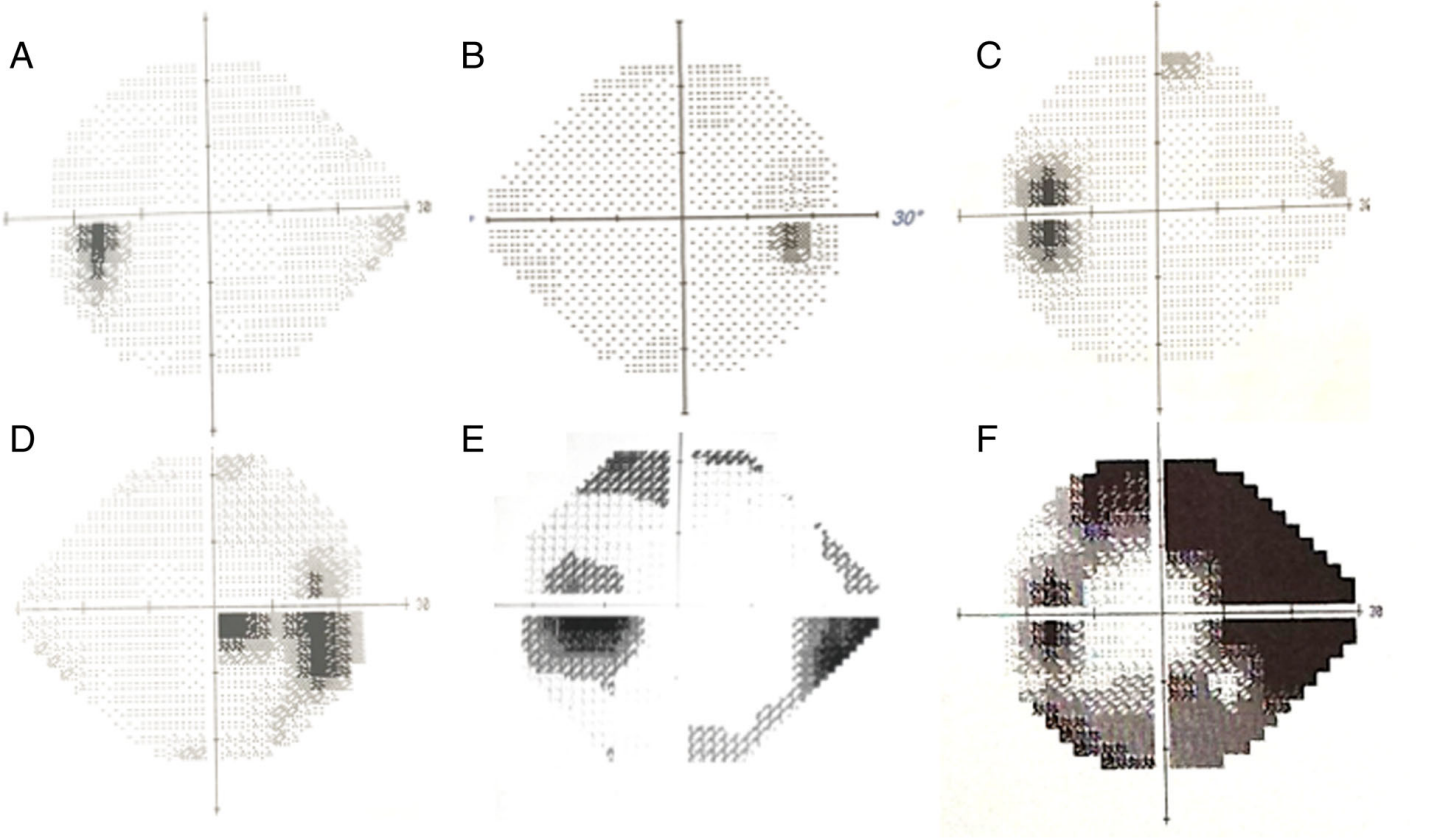
Typical visual field defects in IIH. Common visual field defects seen in IIH with the Humphrey visual field analyser grey scale. **a**, Left eye with a slightly enlarged blind spot; **b**, right eye with slightly enlarged blind spot; **c**, Left eye obvious enlarged blind spot; **d**, right eye with enlarged blind spot and paracentral scotoma; **e**, left eye with enlarged blind spot and prominent inferior nasal step; **f**, Left eye with enlarged blind spot, dense superior and inferior arcuate scotomas

Ophthalmic examination should include: visual acuity, a pupil examination, formal visual field assessment and dilated fundal examination to evaluate the papilloedema. The typical visual field findings are that of an enlargement of the blind spot, peripheral constriction and or an inferior nasal step or partial arcuate defect [22, 29] (Fig. 2). To interpret visual fields with confidence a basic understanding of the plots (Table 2), reliability indices (Table 3), and global parameters (Table 3) is required. Visual field testing is a psychophysical test and can be demanding to interpret in any disease including IIH. Besides physical obstacles, cognitive factors including subject attention, motivation, fatigue, and response bias can influence the obtained thresholds. Improvement in reliability has been shown to improve with clear instructions [30]. Despite clear instructions in the IIHTT, performance failures were observed at some point in one fifth of the participants and were likely due to behavioural factors as the fields returned to baseline values on re-testing: therefore not deemed to be disease progression. Retesting is recommended where there is perimetric worsening, in the presence of unreliable indices (Table 3) [31].

**Table 2:** Interpretation of Humphrey visual field plots. In static perimetry the stimulus is stationary but it changes its intensity until the sensitivity of the eye at the particular point is found. It is measured at preselected locations in the visual field. Most IIH patients have a threshold test where steps of 4 dB are used until detected then re-tested at every point in 2 dB steps


**Term**	**Explanation**	**Notes**
Numerical Display	These are the raw values of the individual’s retinal sensitivity at predetermined points in decibels (dB). Normal values are approximately 30 dB while recorded values of < 0 dB equate to no sensitivity measure.	The HVF analyser uses light between 0 and 50 dB (0 is the brightest and 50 is the dimmest). Sensitivity is greatest in the central field and decreases towards the periphery.
Grey scale	This is a graphical representation of the numerical display. It allows for quick assessment of the field as values closer to 0 dB (low sensitivity) are coded with black and those closer to 50 dB with white (highest sensitivity).	This parameter should not be used alone, the reliability and global indices are critical to interpreting this map.
Total deviation	This demonstrates the difference between measured values and population age-normal values at specific retinal points. The numbers indicate the difference compared to the mean. A negative value indicates less visual sensitivity compared to the mean population.	Both the total deviation and the pattern deviation provide a numerical total plot (top) and the probability plot which gives a visual representation of statistical analysis (t test) of this deviation from the mean; the larger departure from the mean, the darker the symbol.
Pattern deviation	This represents focal depressed areas in the points tested when accounting for overall general reductions of vision caused by media opacities (e.g. cataracts), uncorrected refractive error, reductions in sensitivity due to age and pupil miosis.

**Table 3:** Interpretation of Humphrey visual field parameters

Term	Explanation	Notes	Example
Reliability indices:			
Fixation Losses	Fixation is plotted, if the patient moves and the machine re-tests and patient sees spot then a fixation loss is recorded.	Fixation losses above 20% may significantly compromise the reliability of the test.	OD- Right eye; OS – Left eye. Note the longer the test time the more tired the patient will be.
False POS (Positive) Errors	Patient responds to the normal whirr noise of the computer when it sounds as if is about to present a light but does not.	High false positive score occur in a “trigger happy” patient. < 33% is an unacceptable test.	
False NEG (Negative) Errors	A brighter light is presented in an area in which the threshold has already been determined and the patient does not respond to it.	High false negative score occurs in fatigued or inattentive patients. < 33% is an unacceptable test.	
**Global indices:**			
Glaucoma Hemifield Test (GHT)	This assesses clusters of points above and below the horizontal meridian for any significant difference.	It describes the field as “Within normal limits”, “Borderline” or “Outside normal limits”	24–2 denotes the test strategy (24 degrees temporally and 30 degrees nasally and tests 54 points).
VFI			
Mean deviation (MD)	A measure of overall field loss		
Pattern standard deviation (PSD)	Measure of focal loss or variability within the field taking into account any generalised depression.	An increased PSD is more indicative of glaucomatous field loss than MD.	
**Probability values**	These indicate the significance of the defect < 5%, < 2%, < 1% and 0.5%.	The lower the *p* value the greater its clinical significance and the lesser the likelihood of the defect occurring by chance.	

Reference: Mollan, SP (2018). Investigations and their interpretation. In Denniston AK and Murray PI, 4thed., Oxford handbook of ophthalmology: Oxford University press: Oxford

**Fig. 3:**
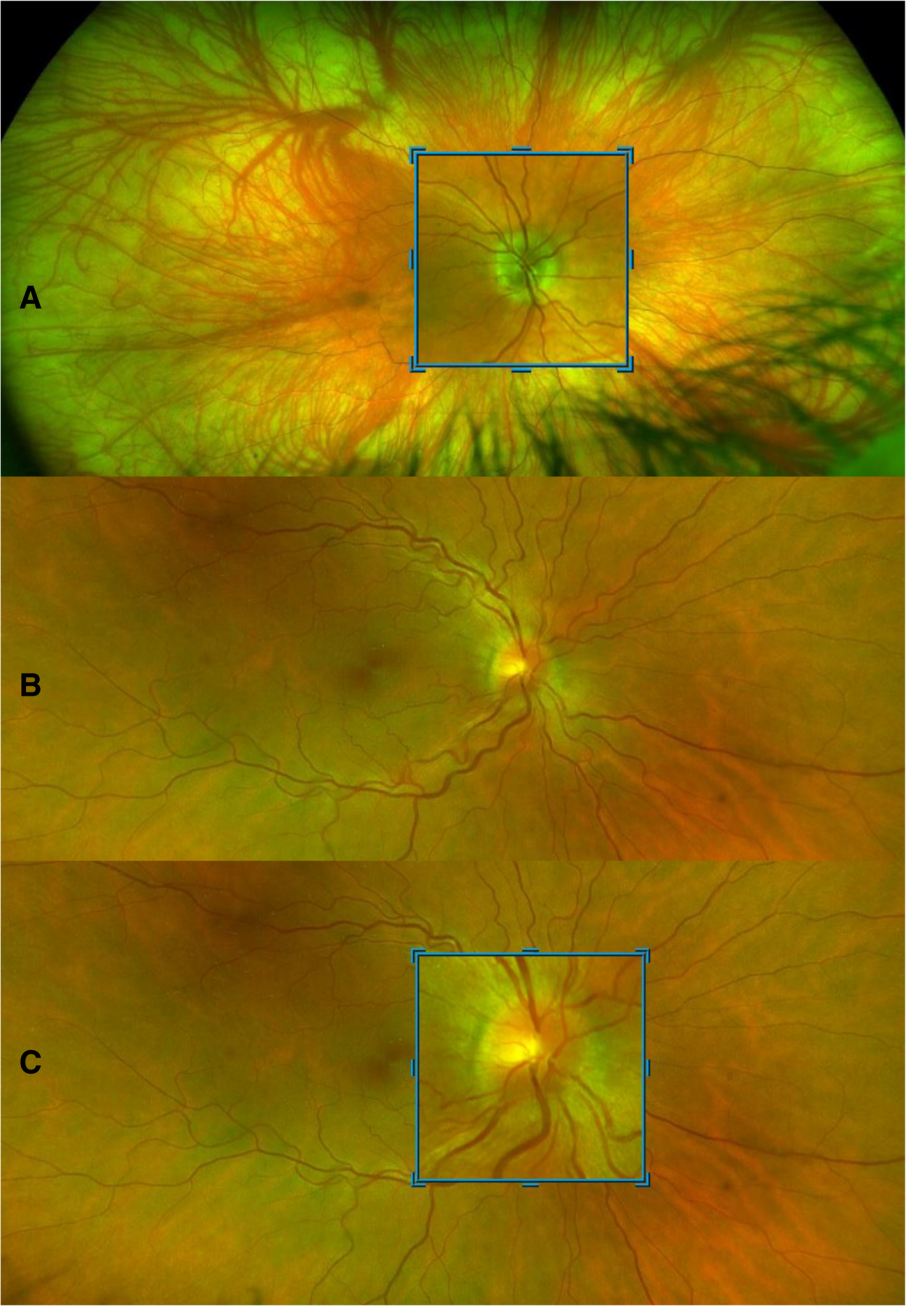
Wide-field imaging using. Wide-field imaging with the Optos™ through an undilated pupil in **a**, normal patient and **b**, a patient with IIH. **a**, normal fundus with blue high magnification box to inspect the optic nerve. Peripapillary atrophy 360^o^ around the disc which is normal. Note the lashes seen inferiorly as artefact on image. **b**, right optic nerve which has grade 2 Frisen swelling where there is elevation of the optic disc margin 360^o^, loss of the clear optic disc margin as seen in **a**. **c**, the high magnification tool allows excellent visualisation of the swelling without degradation of the image

**Fig. 4:**
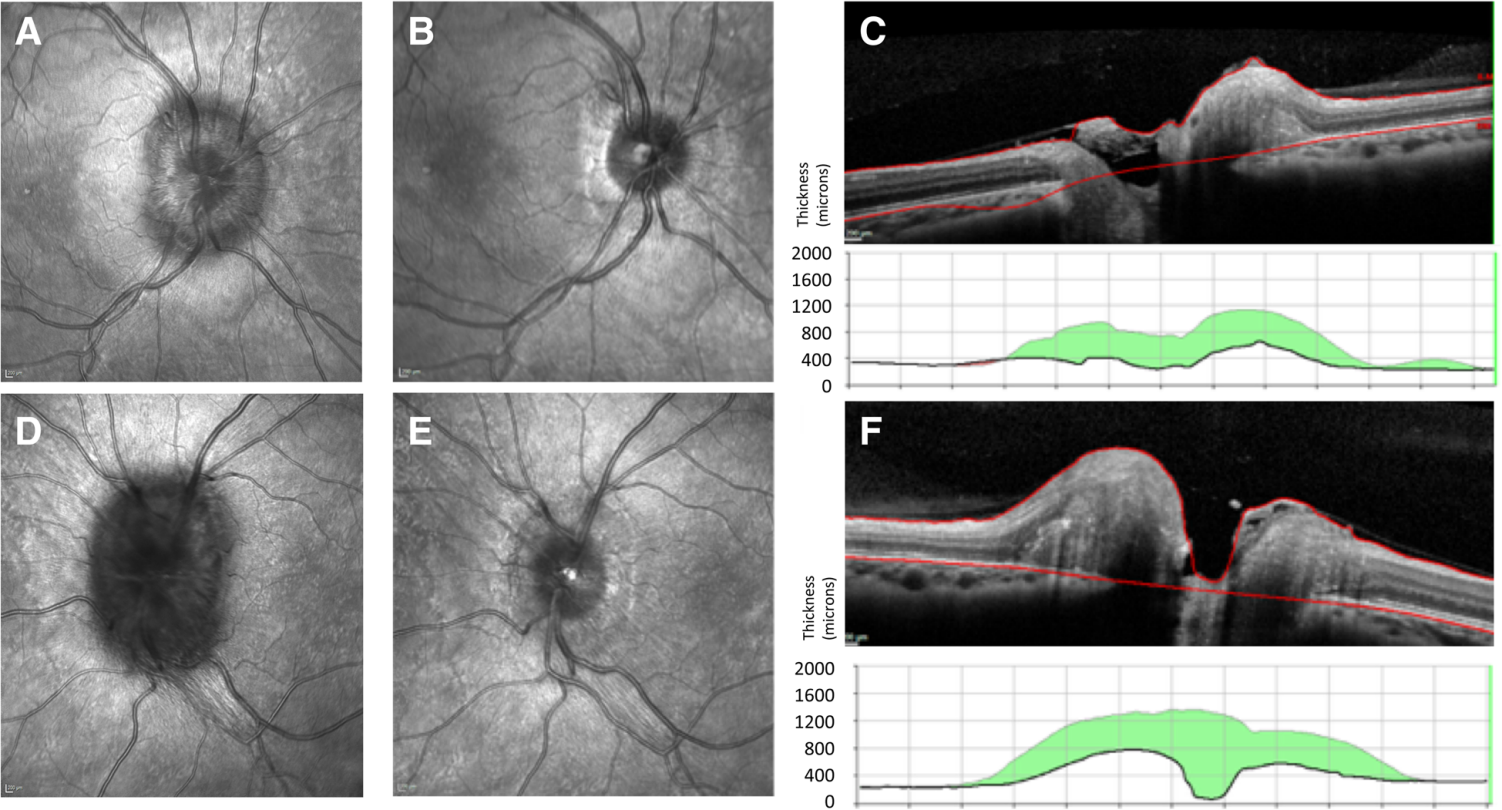
Optical coherence tomography highlighting improvement of papilloedema. OCT is useful for monitoring of changes in papilloedema. **a**, Right eye infrared (IR) image of a swollen optic nerve. Note the Paton’s lines (circumferential lines) between 9 o’clock and 11 o’clock. **b**, Right eye IR image the nerve following a low calorie diet 6 weeks later. Note the tidemark changes of the extent of the previous oedema. **c**, Right eye cross-sectional image half way through the optic nerve head. Note the high line indicates the height of the swelling at diagnosis and the green volume reduction from the first scan to the most recent one (in this case 6 weeks). **d**, Left eye IR image of a swollen optic nerve. Note the difference between **a** and **d**, indicating asymmetric papilloedema with worse papilloedema in the left eye. **e**, Left eye IR image the nerve following a low calorie diet 6 weeks later. Note the tidemark changes of the extent of the previous oedema. **f**, Left eye cross-sectional image half way through the optic nerve head. Note the high line indicates the height of the swelling at diagnosis and the green volume reduction from the first scan to the most recent one (in this case 6 weeks)

**Fig. 5:**
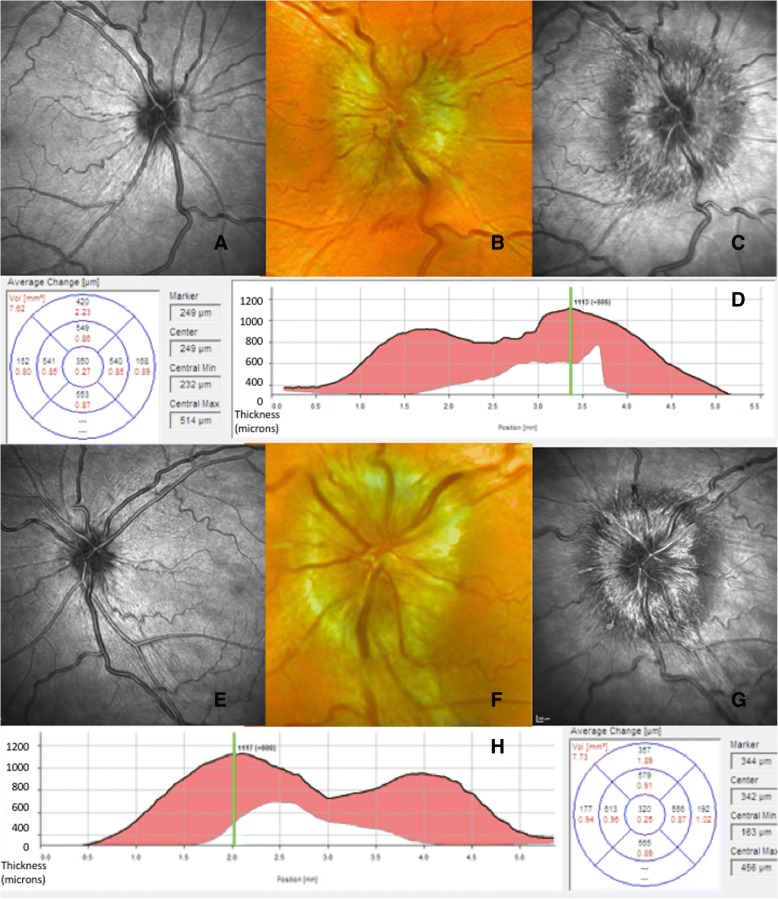
Optical coherence tomography highlighting worsening of papilloedema. OCT is useful for monitoring of changes in papilloedema. **a**, Right eye infrared (IR) image of a normal small optic nerve in a patient in IIH with ocular remission. Note the tidemark changes of the extent of the previous oedema. **b**, Colour photograph of right optic nerve with swelling and haemorrhage with recurrence of symptoms. **c**, Right eye IR image taken at the same time as **b**. Note the extent of the oedema and the optic nerve is more visible with the OCT image compared to the photo. **d**, Right eye cross-sectional image half way through the optic nerve head. Note the high line indicates the height of the swelling at this visit and the red volume increase is from the last OCT scan to the most recent one. **e**, Left eye IR image of a normal small optic nerve in a patient in IIH with ocular remission. Note the tidemark changes of the extent of the previous oedema. **f**, Colour photograph of left optic nerve with swelling and cotton wool spot changes with recurrence of symptoms. **g**, Left eye IR image taken at the same time as **f**. Note the extent of the oedema and the optic nerve is more visible with the OCT image compared to the photo. **h**, Left eye cross-sectional image half way through the optic nerve head. Note the high line indicates the height of the swelling at this visit and the red volume increase is from the last OCT scan to the most recent one

The role of imaging the optic nerve head is becoming increasingly important. Qualitative analysis of photographic images remains useful for clinical records. Newer techniques such as wide field imaging using the Optos™ allow high resolution image capture through an undilated pupil, with a magnification tool to examine the optic nerve head (Fig. 3). Optical Coherence Tomography (OCT), in particular spectral domain OCT, offers a non-invasive technique for both qualitative and objective quantification of papilloedema that can support the detection of papilloedema [32–34]. OCT is found to be a valuable tool for quantification of papilloedema in longitudinal assessment: Fig. 4 demonstrates the clear improvement in papilloedema in a newly diagnosed IIH patient who undertook a low calorie diet for 6 weeks [35]. However, OCT has limitations. In cases of severe papilloedema errors occur in the automated software analysis which can lead to unreliable values, these scans need manual adjustment to ensure reliable results [36]. However as peripapillary retinal nerve fibre layer (RNFL) thickness resolves, most systems do not allow the distinction between reduction in oedema and optic atrophy as both conditions lead to a reduction in RNFL thickness [37]. Software enhancements on certain OCT platforms allow the determination of ganglion cell layer thickness and this may prove to be beneficial to correlate with visual loss. Some OCT platforms have the ability to capture short video footage which is useful to document location and presence of spontaneous venous pulsations (Additional file 1). OCT imaging supports the diagnosis and monitoring of papilloedema as demonstrated in Fig. 5 where there is an increase in the volume at the optic nerve head with recurrence of symptoms of headache and pulsatile tinnitus for one month in a patient previously in ocular remission, but does not mitigate the continued monitoring of all measures of visual function through clinical examination and formal perimetry.

#### Other symptoms

IIH can cause other symptoms, as well as headache and visual disturbances. Recent data however suggests that other systems may also be affected in IIH, presumably as the result of increased ICP, although the exact mechanisms leading to these alterations remain largely unknown. In this context, structural changes in the olfactory nerve [38], which are associated with an olfactory dysfunction in up to 80% of IIH patients, have been demonstrated [39, 40]. Nevertheless, despite an easy determination of olfactory function by the use of extended Sniffin’ Sticks, this observation is not useful for the diagnosis or follow-up examinations in IIH as the effects of a variation in ICP may reflect on olfactory function with a significant delay.

A uni- or bilateral pulsatile tinnitus is commonly observed in IIH [7, 8, 41]. Its underlying cause is not entirely elucidated but it is hypothesized that stenoses in the transverse sinus, which are commonly observed in IIH, may induce audible turbulences in blood flow. Nevertheless further studies are required to confirm this assumption.

Unilateral or bilateral sixth-nerve palsy may occur in IIH causing horizontal diplopia [42, 43]. The reason for this neurological deficit in the context of IIH has not been fully investigated. The pathophysiological mechanism has traditionally been thought to be due to compression against the petrous ligament or the ridge of the petrous temporal bone, or stretching along the intracranial course of the nerve.

Cognitive function has been reported to be affected in IIH. While a number of small uncontrolled studies, which occasionally focused only on single neuropsychological domains, suggested a relationship between IIH and cognitive decline [44–46], Yri et al. demonstrated in an extensive prospective case-control study that IIH is associated with a global cognitive dysfunction with the most extensive deficit in reaction time and processing speed [47]. Interestingly, the results of this study reveal that despite an improvement of ICP and headache after 3 months of adequate treatment, the cognitive dysfunction appears to persist raising the question if IIH-related cognitive decline is the result of more complex mechanisms rather than the direct effect of mechanical compression. These neuropsychological deficits may hamper significantly a professional reintegration after prolonged absence from work.

### Investigations

#### Neuroimaging

Brain imaging is an essential part of the diagnostic algorithm and MRI should be considered the gold standard of care to exclude secondary causes of elevated ICP and to identify structural alterations associated with IIH. These include an empty sella turcica (or at least significant changes in size, shape and volume of the pituitary gland) and a flattening of the posterior optic globe. IIH also leads to an enlargement of the optic nerve sheath and an increased tortuosity of the optic nerve [12, 48–51]. However, the volume of the optic nerve is not affected [12]. In contrast to early imaging studies based on plain film X-ray imaging that suggested a reduced ventricle size (slit-like ventricles) as indicative for IIH [52], data based on CT and MR imaging techniques demonstrates that size and volume of the lateral ventricles are not altered in IIH [49, 53].

Diagnostic brain imaging in IIH should always include a CT- or MR venography to exclude a venous sinus thrombosis as even clinically inapparent microthromboses may induce a venous outflow obstruction that increases ICP [54]. Furthermore the venography may demonstrate the presence of uni- or bilateral transverse sinus stenoses (TSS) as these are frequently observed in IIH patients with reported prevalence rates of up to 90% [51, 55, 56]. However, if TSS are cause or consequence of elevated ICP remains controversial. Additionally, asymmetry of the transverse sinus can occur in 50% of healthy individuals [57].

Despite the value of neuroimaging in the diagnostic workup of IIH, it does not replace the need for a measurement of lumbar opening pressure as imaging abnormalities show a large interindividual variation and none of the findings are pathognomonic of IIH hence imaging findings only serve as supportive evidence for the diagnosis of IIH.

#### Lumbar puncture

LP is mandatory in the diagnostic algorithm of IIH. In addition to a normal CSF composition, diagnostic criteria require that opening pressure, which should be measured in the lateral decubitus position with stretched legs and without sedative medications, should not exceed 25 cmH_2_O in adults and 28 cmH_2_O in children [2, 58, 59]. Increases of ICP may also occur intermittently, in particular in IIH patients without papilloedema [60, 61]. Therefore, if IIH is suspected but opening pressure lies within the normal range during the initial assessment, a second measurement or even continuous monitoring could be considered [61–63]. Conversely, pressures above 25 cmH_2_O can occur in normality [64], with pressures in the range 25–30 cmH_2_O constituting a grey area that could be pathological or normal and should be interpreted with caution [43]. Patients with pressures in this “grey zone” should be evaluated cautiously to ensure they meet the other aspects of the IIH diagnostic criteria [43].

#### Blood tests

Blood tests should be performed for the exclusion of secondary causes of elevated ICP or other medical conditions that may clinically present with similar symptoms. Blood test should be tailored to the individual patient’s presentation. In the absence of typical phenotypic characteristics of IIH (obese female of childbearing age) blood tests to exclude secondary causes of pseudotumor cerebri are key. Excluding conditions that increase the likelihood of a sinus vein thrombosis, which could obstruct venous outflow, may be performed. These could include analysis of coagulation parameters to identify hypercoagulable states as well as a search for diseases that are associated with a higher risk of venous (micro-) thrombosis such as systemic lupus erythematosus and infections of the middle ear or mastoid. Endocrine disorders that may mimic IIH symptoms include Addison’s disease, Cushing’s syndrome, hypoparathyroidism as well as the use of growth hormones and may need to be excluded. Other medical conditions that may induce a secondary increase of ICP and should be identified via serologic examinations include systemic infections, uraemia, renal failure and anaemia [65] (Table 4).

**Table 4:** Medical conditions that may induce a secondary elevation of ICP or produce symptoms that may mimic IIH (adapted from [2, 25, 66–69])

*1. Medical disorders that may induce a sinus vein thrombosis or that may cause a venous outflow obstruction through other mechanisms:* • Thrombophilia and other hypercoagulable conditions • Systemic lupus erythematodes • Infections of the middle ear or mastoid • CNS- infections • Increased right heart pressure with pulmonary hypertension • Chronic obstructive pulmonary disease • Superior vena cava syndrome • Arteriovenous fistulas • Glomus tumour • Tumour process that may compress parts of the venous outflow system *2. Medications* • Fluoroquinolones [70] • Tetracycline • Vitamin A and retinoids • Anabolic steroids • Withdrawal of corticosteroids (in particular after prolonged administration) • Administration of growth hormone • Lithium • Nalidixic acid • Oral contraceptives • Levonorgestrel implant system • Amiodarone • Cyclosporine • Cytarabine *3. Other medical conditions* • HIV • Syphilis • Borreliosis • Varicella • Addison’s disease • Hypoparathyroidism • Obstructive sleep apnoea • Pickwickian syndrome • Uraemia • Severe iron deficiency anaemia • Renal failure • Turner syndrome • Down syndrome	

## Treatment

### Surgical treatment

Surgical management is essential for IIH patients with rapidly declining visual function. The evidence base for choice of surgical technique is lacking and practice varies internationally and with surgeon preference. CSF diversion procedures including ventriculo-peritoneal, lumbo-peritoneal, and less frequently ventriculo-atrial shunting may be utilised. Ventriculo-peritoneal shunts are preferred due to lower revision rates compared to lumbo-peritoneal shunts (1.8 versus 4.3 revisions per patient respectively) [66]. Ventriculo-peritoneal shunts are typically placed using neuro-navigation and adjustable valves (anti-gravity or anti-siphon devices) that can reduce the risk of low pressure headaches [66]. However, ventriculo-peritoneal shunt insertion leads to a temporary driving restriction in some countries such as the United Kingdom. Lumbo-peritoneal shunting may be considered but should be avoided in those with low lying cerebellar tonsils due to the risk of post-operative cerebellar tonsillar descent. Shunt revision is common with 51% requiring revision and multiple revisions required in 30% [67]. Complications can occur including abdominal pain, shunt obstruction, migration and infection, low pressure headaches and subdural haematoma [67, 68]. An alternative to shunting is optic nerve sheath fenestration (ONSF) [69] which is more cost effective in some health care systems than CSF shunting [66]. But, this procedure also has a 26% revision rate, due to closing over of the fenestration, with an ensuring rise in ICP and consequent potential for further visual deterioration [66]. Headache improvement after ONSF is variable (one third to one-half have no headache response) [70].

The Neuro-Ophthalmology Research Disease Investigator Consortium (NORDIC) are currently planning a randomised controlled surgical trial, SIGHT, which will compare shunting with ONSF and acetazolamide. The trial will recruit patients with a severe visual loss defined as a parametric mean deviation between -6 dB and -27 dB assessed on the Humphrey visual field analyser.

### Endovascular stenting

Venography brain imaging in IIH frequently demonstrates venous sinus stenosis [55, 71]. These stenoses typically regress after CSF drainage which induces reduction of ICP, consequently the stenoses are thought to represent an effect of raised ICP not the underlying cause [72]. The extent of the stenoses does not correlate with ICP or predict the risk of visual loss [55]. Some centres are conducting venous sinus stenting to treat IIH but utility is debated. Case series have reported improvement in symptoms of intracranial hypertension, however case selection is not randomised which can lead to selection bias and there are a lack of long term outcomes [73]. Complications of the procedure are reported and include a short-lived ipsilateral headache in many, stent-adjacent stenosis that requires retreatment in a third, and in rare cases vessel perforation leading to acute subdural haematoma, stent migration, thrombosis and death [73]. The comparative efficacy of stenting and shunting is not established, nor are the long-term efficacy, revision rate and safety data. There may be a role in some highly selected IIH patients [74].

We do not advocate CSF diversion or shunting techniques to treat isolated headache symptoms due to the poor outcomes (ongoing headache in 68% at 6 months, 77% at 12 months and 79% at 2 years post-shunting), high revision rates and risk of complications [67]. There is insufficient evidence to support venous stenting to exclusively treat headache.

### Disease modification through weight loss

There is a clear association between IIH and weight with over 90–95% of patients being obese [4]. Additionally, IIH is reported in the context of gaining 5–15% of body weight [75]. Weight loss is the only established disease modifying therapy in IIH [76]. Consequently, patients should be sensitively counselled about the importance of weight loss. However, the amount of weight loss required is not well established. Additionally, the optimal method of weight loss is uncertain. Dietary strategies are notoriously difficult to achieve and maintain in the long term [77, 78]. The role of bariatric surgery is being increasingly suggested as a lasting therapy to induce IIH remission.

Bariatric surgery leads to greater weight loss compared to dietary regimes, with mean reduction in body mass index (BMI) of 7.05–15.34 m/kg^2^ at 12 months [79] and significant sustained long term weight loss [80]. Bariatric surgery has been found to be cost effective for other obesity related conditions with very low associated mortality (0.05%–0.14%, which is akin to laparoscopic cholecystectomy) [79, 81, 82]. A systematic review of the IIH cases treated with bariatric surgery report 100% resolution of papilloedema and 90% experience headache improvement [83]. A randomised control trial evaluating bariatric surgery in IIH is underway (IIH Weight Trial) [84].

### Symptomatic therapy with therapeutics

#### Acetazolamide

Therapeutic agents currently used in IIH aim to reduce ICP through reduction in CSF secretion. There are few therapeutic options and the recent Cochrane review reported: “the two included randomised controlled trials showed modest benefits for acetazolamide for some outcomes, there is insufficient evidence to recommend or reject the efficacy of this intervention, or any other treatments currently available, for treating people with IIH [85]”. The IIHTT is the largest RCT to date and reports improvement in visual field function in patients with mild visual loss when treated with acetazolamide [86]. Benefits were most marked in those with the most marked papilloedema. However high doses of acetazolamide were used (greater than 40% of patients were treated with 4 g of acetazolamide daily) and this may not be tolerable. Previous studies have demonstrated that 48% of patients discontinue acetazolamide when doses of just 1500 mg are utilised [87]. Side effects include paraesthesia, dysgeusia, vomiting and diarrhoea as well as malaise, fatigue and depression [88, 89]. Acetazolamide is the only therapeutic that has been evaluated in RCT and is regarded as the first line therapy for IIH. However, not all clinicians prescribe acetazolamide for IIH due to the limitations of the evidence base highlighted by the 2015 Cochrane review in conjunction with the potential side effect profile.

In pregnancy the use of acetazolamide is controversial. Data from case series (*n* = 50 IIH patients using acetazolamide in the first trimester) has not identified an increased risk of foetal malformations although animal data has highlighted teratogenic effects [90, 91].

#### Alternative therapeutics

A number of alternative therapeutic agents are used in IIH, however there is little evidence to support their use.

In animal studies, intravenous and intraventricular high dose furosemide has been shown to reduce CSF secretion by 20–50% [92–95]. However, clinically relevant routes of delivery and dosing have not been studied. A paediatric case series (*n* = 8) demonstrated that together acetazolamide and furosemide reduced ICP over 6 weeks, however the absence of a control group limits interpretation [96]. Bumetamide does not reduce CSF secretion in animal studies, and human studies have not been conducted [92].

Amiloride administered into the carotid artery in animal models reduces CSF secretion by up to 50% [97, 98]. But these studies have not used clinically relevant doses or routes of delivery.

Octreotide has been hypothesised to manipulate CSF secretion as there are somatostatin receptors on the choroid plexus [99]. A prospective open-label study of 26 patients reported resolution of papilloedema in 92% of cases however cautious interpretation is needed in the absence of a control group [100].

Topiramate utility in IIH has been suggested by an open label study which randomly assigned 40 patients to acetazolamide or topiramate. They demonstrated treatment equivalence with all patients experiencing improvement in visual fields (ICP was not measured) [101]. Topiramate may have additional advantages in IIH as it can induce weight loss and has efficacy as a migraine preventive therapy [102–105]. Recently reported in vivo studies demonstrated that both subcutaneous and oral administration of topiramate significantly lowers ICP in rodents whilst other drugs tested, including acetazolamide, furosemide, amiloride and octreotide, did not significantly reduce ICP [106].

#### Novel therapeutics

Future therapies would ideally control ICP acutely as well as treat the underlying disease process through weight loss. There is growing interest in the role of gut neuro peptides in IIH. The gut peptide glucagon-like peptide-1 (GLP-1) regulates insulin secretion and weight, and currently GLP-1 mimetic drugs are used extensively to treat diabetes (without risk of hypoglycaemia) and obesity [107]. Recent in vitro assays have demonstrated that the GLP-1 receptor agonist exendin-4 reduces CSF secretion [108]. Additionally, clinically relevant doses of exendin-4 dramatically reduced ICP in rodents with raised ICP (44% reduction in ICP within 10 min of dosing with effect maintained for 24 h [108]. A clinical trial is currently underway exploring the physiological effects of exenatide in reducing ICP in IIH.

Therapeutic agents inhibiting the actions of 11Beta hydroxysteroid dehydrogenase type 1 (11β-HSD1) have been proposed in IIH. 11β-HSD1 is an enzyme which converts inactive cortisone to active cortisol and consequently regulates local cortisol availability, a key determinant of fluid secretion [109]. 11β-HSD1 inhibitors have been shown to reduce intraocular pressure through reduction of aqueous humour production by the ocular ciliary body [110]. Akin to this mechanism, 11β-HSD1 is functionally active in the CSF secreting choroid plexus epithelial cells [111]. In patients with IIH, reduction in ICP correlates with reduction in global 11β-HSD1 activity measures [111]. 11β-HSD1 inhibitors have been developed to treat obesity and metabolic syndrome. A phase 2 randomised controlled trial has been conducted in IIH which assesses a specific 11β-HSD1 inhibitor (AZ4017), results are awaited [112].

### Managing headache

Headache is the predominant morbidity in IIH and significantly reduces quality of life [9]. Headaches occur not only during the active stages of the disease when ICP is elevated, but frequently continue even after the ICP has settled [10, 67]. In those IIH patients with resolved papilloedema (IIH in ocular remission), the ongoing headaches cause significant morbidity. Evidence to guide headache management is very limited. Principles of managing headache in IIH could include [113]:

1. In those IIH patients with ongoing raised ICP, weight loss has been shown to significantly improve the Headache Impact Test 6 score (HIT-6) as well as headache severity, frequency and acute analgesic use [114]. Interestingly acetazolamide does not improve headache disability scores on the HIT-6 [86].
2. Medication-overuse headaches are a frequent co-morbidity in IIH (37%) and patients will likely benefit from withdrawal [11].
3. Headache phenotype should be carefully evaluated to look for features of co-existing migraine. In those with IIH in ocular remission and migraine or in those with migraine-like headache with active IIH, acute and preventive strategies may be useful although there are no dedicated studies in this area. Preventive strategies may have particular benefit for those patients in whom the ICP is settling (IIH in ocular remission). Choice of migraine prevention should consider avoiding drugs that induce weight gain. Potential choices could include topiramate, candesartan and onabotulinum toxin A, although there is no evidence of efficacy of these drugs in the setting of IIH [113, 115].

### Therapeutic lumbar puncture

Therapeutic serial LPs are not advocated as a long-term treatment strategy for IIH. Although LP induces a transient reduction of CSF pressure the effect is typically short lived with pressures rising rapidly after the procedure [116]. Therapeutic LP has limited application for managing headache, as headache improves in 71%, but the improvement is small (1 point on the verbal rating score 0–10) and there is also a 64% chance of a headache exacerbation in the week following LP in IIH patients [117]. Additionally, IIH patients frequently report a very negative and emotional experience when they undergo a LP [118, 119]. In the short term, LP may have a role as a temporising procedure to preserve vision in patients with fulminant IIH awaiting an imminent CSF diversion procedure.

### Pregnancy

Pregnancy can potentially limit the ability to perform neuroimaging studies for investigation and restrict therapeutic options. Hence the management of those who either present in pregnancy for the first time with IIH or for the majority who become pregnant during the course of their disease management should be determined on a case-by-case basis [120–122].

Patients should be cautioned about excessive weight gain during pregnancy that could precipitate a worsening of their IIH. If there is access to a weight management service, this is useful so that clear advice regarding weight gain is appropriate for the gestational age of the foetus [123].

Use of acetazolamide in pregnancy is controversial, and should be discussed with the individual as perinatal exposure in rodents has caused teratogenic effects [124, 125]. The postaxial limb deformities, such as polydactyly or limb deficiency, that have been reported in small animals were not found in primate studies [126]. The manufacturers do not recommend its use in pregnancy, but some have reported using it in other conditions and the risk of malformation was no higher than expected in the general population however few patients had exposure in the first trimester [127]. In the largest study on the use of acetazolamide in IIH, 101 pregnant women (158 pregnancies) took acetazolamide in a daily dose ranging between 250 and 2000 mg. Over half took acetazolamide prior to 13th gestational week, abortion rate (both spontaneous and induced) was higher in the acetazolamide group; the authors therefore recommended that acetazolamide should be used with caution and justification in the pregnant IIH patient [91].

Topiramate should not be used in pregnancy, due to the clear higher rate of foetal abnormalities following its use [128]. Many other headache treatments are not recommended in pregnancy; therefore the risk-benefit should be discussed with the patient.

Few patients present with IIH in pregnancy, and if there is imminent risk to the vision, serial LPs, optic nerve sheath fenestration or CSF diversion can be considered [120, 129]. Clear communication with the obstetric and gynaecological services is key to help reassure healthcare professionals and the patient. Increased observations during pregnancy also serves to reassure. Alterations to birth plans should not be made on the basis of the historical diagnosis of IIH and only if there is likely to be precipitous visual decline in the settling of moderate to severe papilloedema. If there is a potential for a prolonged second stage of labour assisted deliveries (such as caesarean section or instrumental deliveries) should be considered.

### Idiopathic intracranial hypertension without Papilloedema (IIHWOP)

This is a rare form of IIH where there is no evidence of papilloedema in the setting of raised ICP. Headache is the principle morbidity in these patients.

The diagnostic criteria for definite IIHWOP are the same as for IIH, except there is no papilloedema, but unilateral or bilateral sixth nerve palsies may be present. A diagnosis of possible IIHWOP can be made without the presence of unilateral or bilateral sixth nerve palsies, however in this case at least 3 out of the following 4 features on brain imaging need to be present: an empty sella, flattening of the eye globe, widening of the space around the optic nerve and/or transverse venous sinus stenosis [2]. In patients with IIHWOP risk of vision loss has not been identified and does not seem to develop over the disease course. The commonest symptoms of IIHWOP include headache, pulsatile tinnitus, visual phenomena such as visual obscurations, blurred vision, photopsia and diplopia (due to sixth nerve palsy) [27].

Once a diagnosis of IIHWOP is established, all patients should be counselled about weight management. As there is no threat to vision, long term visual monitoring is not required. Management of headache should be the same as in IIH. Escalation of management to surgery to control elevated ICP in IIHWOP should not be routinely considered.

## Conclusion

IIH is a challenging disease which crosses many specialties. At investigation it requires careful exclusion of secondary causes through history, neuroimaging, LP and ophthalmic examination. Once a diagnosis is established of typical IIH, it requires regular visual monitoring, neurological input for active headache management, and direct conselling regarding weight loss. When there is no immediate threat to vision, medical treatment with acetazolamide should be considered. Less commonly required are surgical treatments to preserve vision when fulminant IIH exists. There is increasing research in this area and as evidence is published this document will require timely revision.

## Additional file

Additional file 1: This is an infrared 10 s video as taken with the Heidelberg Engineering™ optical coherence tomography instrument. There is clear evidence of spontaneous venous pulsation at the optic nerve head in a normal person. The veins at the optic nerve head cup move in and out, with colour and shape change. (MOV 4666 kb)

## Abbreviations

11β-HSD1: 11Beta hydroxysteroid dehydrogenase type 1
BMI: Body Mass Index
CSF: Cerebrospinal fluid
CT: Computerised tomography
GLP-1: Gut peptide glucagon-like peptide-1
ICHD: International Classification of Headache Disorders
ICP: Intracranial pressure
IHS: International Headache Society
IIH: Idiopathic intracranial hypertension
IIHTT: Idiopathic Intracranial Hypertension Treatment Trial
IIHWOP: Idiopathic intracranial hypertension without papilloedema
LP: Lumbar pincture
MR: Magnetic resonance
MRI: Magnetic resonance imaging
NORDIC: Neuro-Ophthalmology Research Disease Investigator Consortium
OCT: Optical coherence tomography
ONSF: Optic nerve sheath fenestration
RCT: Randomised controlled trial
RNFL: Retinal nerve fibre layer
